# Free Space Detection Algorithm Using Object Tracking for Autonomous Vehicles

**DOI:** 10.3390/s22010315

**Published:** 2021-12-31

**Authors:** Yeongwon Lee, Byungyong You

**Affiliations:** Department of Mechanical Engineering, Kyungil University, Gyeongsan 38428, Korea; ywuu1214@gmail.com

**Keywords:** autonomous vehicle, free space detection, object tracking, LiDAR sensor

## Abstract

In this paper, we propose a new free space detection algorithm for autonomous vehicle driving. Previous free space detection algorithms often use only the location information of every frame, without information on the speed of the obstacle. In this case, there is a possibility of creating an inefficient path because the behavior of the obstacle cannot be predicted. In order to compensate for the shortcomings of the previous algorithm, the proposed algorithm uses the speed information of the obstacle. Through object tracking, the dynamic behavior of obstacles around the vehicle is identified and predicted, and free space is detected based on this. In the free space, it is possible to classify an area in which driving is possible and an area in which it is not possible, and a route is created according to the classification result. By comparing and evaluating the path generated by the previous algorithm and the path generated by the proposed algorithm, it is confirmed that the proposed algorithm is more efficient in generating the vehicle driving path.

## 1. Introduction

In order for an autonomous vehicle to travel stably in various environments, it is important to recognize obstacles around the vehicle. In particular, in an environment in which lanes are not recognized while driving or in a road environment in which a precise map is not built, a path must be created by detecting an obstacle-free space, that is, a free space to generate a driving path. Many papers deal with free space detection. In previous papers, there are many cases of detecting free space using only the location information of obstacles. In this case, the behavior of the surrounding obstacles cannot be predicted, meaning there is a high possibility of creating an inefficient path in terms of safety.

Assume that there is an obstacle traveling in the opposite lane of the traveling vehicle. In this environment, if the free space is detected in units of frames using only the location information, it will be detected as a free space that can travel up to the opposite lane area because it does not know which direction the obstacle is traveling in. In this case, there is a possibility of creating a driving route in the opposite lane, and the collision risk is increased. If it is possible to know at what speed and direction the surrounding obstacles are moving with respect to the driving vehicle, then more efficient path generation will be possible. 

In this paper, to solve this problem and increase the efficiency of path generation in free space, we propose a new algorithm for detecting free space based on the speed information of obstacles. The data were acquired using a LiDAR (light detection and ranging) sensor mounted on the actual vehicle, the speed of the obstacle was calculated through the object tracking process, and the free space was divided into four areas according to the speed. For comparison with the previous algorithm, a driving route was generated according to the free space detection result. By comparing the path generated by the previous method and the proposed method, it is confirmed that a more efficient path is generated with the proposed method.

Recently, various studies on autonomous vehicles have been conducted in various fields. There are papers on various signal processing methods of LiDAR sensors mainly used in autonomous vehicles [1,2,3,4,5,6,7,8,9,10,11,12,13,14,15,16,17,18,19,20,21,22,23,24]. Among them, there are many papers that presented research on free space sensing. Free space detection is being studied not only for the study of autonomous vehicle driving, but also in various fields such as the recognition of a parking space or the driving of an aircraft [25,26,27]. Among them, the research trends in the detection of free space in autonomous vehicles are as follows [1,2,3,4,5,6,7,8,9,10,11,12]. Often, a camera or LiDAR sensor is used, or both sensors are used, to recognize the space and obstacles around the vehicle. These sensors recognize the surrounding space and detect a drivable free space free from obstacles. In this process, existing papers often detected empty space using only the location data of obstacles. H. M. Eraqi et al. [1] used a laser scanner to recognize the surroundings and process signals in units of frames to understand the surrounding environment of a driving vehicle. Using these static data, the ground and obstacles are recognized, and the free space is expressed in a simplified polygonal form. C. Fernandez et al. [2] used LiDAR and camera sensors to detect free space and speed bumps. This method detects based on static data and does not take into account the speed of obstacles. J. Tao [3] used LiDAR sensor data to detect free space, in which free space was also detected without information about the speed of the obstacle. Many previous studies used static data without considering the speed of obstacles [1,2,3,4,5,6,7,8,9,10,11,12]. Free space detection for autonomous driving must also be considered in dynamic environments. To supplement this, in this paper, we propose a method to detect free space by reflecting the velocity data of obstacles.

Table 1 summarizes existing papers [1,2,3,4,5,6,7,8,9,10,11,12]. In previous papers, sensors were used in the FSD process, and the speed information of obstacles was summarized. In [1,2,3,4,5,6,7,8,9,10,11,12], obstacles were mainly recognized using LiDAR or camera sensors, and all of them detected free space without information on the velocity of the obstacles.

## 2. Materials and Methods

### 2.1. Clustering and Object Tracking

**Figure 1:**
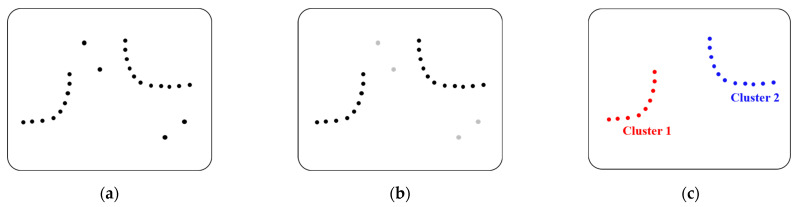
DBSCAN clustering: (**a**) raw data of LidAR sensor that recognized two obstacles; (**b**) data with noise removed; (**c**) data of two clustered obstacles.

This is the process of identifying the behavior of obstacles around the vehicle using sensor data. LiDAR is an abbreviation of light detection and ranging. It is a radar system that measures the positional coordinates of a reflector by measuring the time it takes to emit a laser pulse, reflect it, and return it, which are output as point data. The point data output by the LiDAR sensor are clustered using the DBSCAN (density-based spatial clustering of applications with noise) algorithm [28,29,30]. DBSCAN is one of the clustering algorithms based on density and is a widely used algorithm for clustering point data. Figure 1 is a pictorial representation of the DBSCAN clustering process. Figure 1a shows the single-layer LiDAR raw data. Figure 1b is the result of judging data having a number of points greater than or equal to the MinPts (minimum number of points) in the eps (epsilon) radius as cluster data according to the DBSCAN algorithm, and separating the rest into noise data. Figure 1c is the result of clustering into Cluster 1 and Cluster 2 after removing noise.

**Figure 2:**
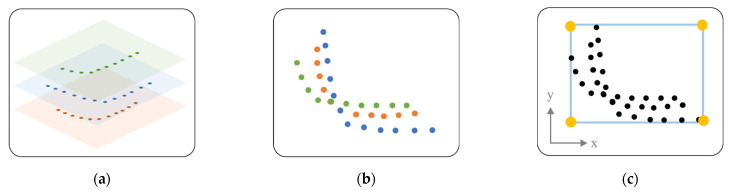
Multi-layer clustering: (**a**) classification of data that recognize the same obstacle in each layer, and 3 single layers that recognize the same obstacle are shown in 3D; (**b**) representation of all 3D data on a 2D plane; (**c**) representation as square data for data simplification.

The boxing process is executed in multiple layers using the results of clustering in a single layer. This is to integrate and simplify multi-layered data. This process is shown in Figure 2. Figure 2a is a classification of data that recognize the same obstacle in each layer, and 3 single layers that recognize the same obstacle are shown in 3D. For integration, the data are integrated and displayed in 2D on one plane as in Figure 2b. For the simplification of the integrated data, square data are created with four points, as shown in Figure 2c. The rectangle is calculated using the x max, x min, y max, and y min values of the integrated point data. As a result, cluster data of multiple layers are represented by four-dot square data.

**Figure 3:**
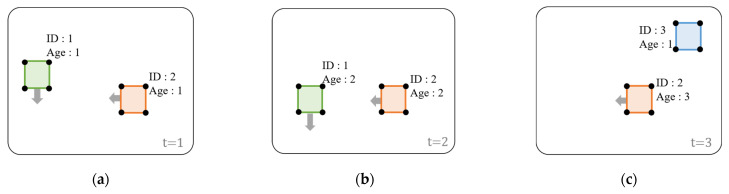
Object tracking. Obstacle position change with time (t) change: (**a**) examples of obstacles when t = 1; (**b**) examples of obstacles when t = 2; (**c**) examples of obstacles when t = 3.

An object tracking process is required to determine the speed of the obstacles [31]. Figure 3 shows the object tracking process. By comparing the previous and current positions of obstacles, it is judged whether they are the same obstacles, and an ID and Age are assigned to each obstacle. If it is judged to be the same obstacle, the same ID as before is assigned and the Age is increased. If it is determined that it is a newly measured obstacle, a new ID is assigned. Through this process, the speed can be calculated using the displacement according to the time the obstacle moves. In this case, when the position of the obstacle is changed, the data of the rectangle may also be changed. Therefore, the minimum and maximum sizes of obstacles (mainly vehicles) are reflected and tracked.

### 2.2. Grid Map

**Figure 4:**
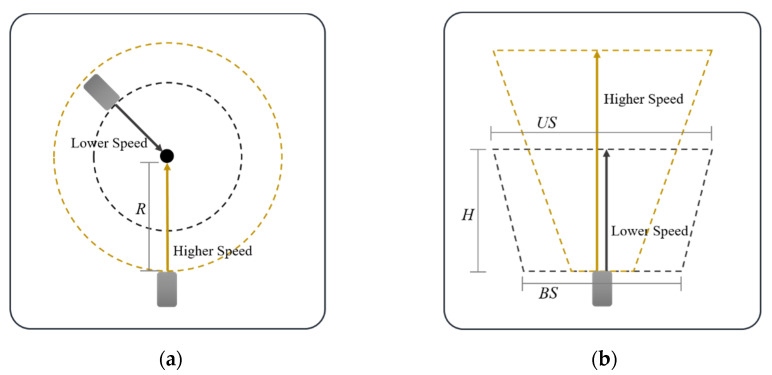
(**a**) Circular grid map of previous paper [1]. (**b**) Proposed trapezoidal grid map. R is the radius of the circle. US is the upper side of the trapezoid. BS is the base of the trapezoid. H is the height of the trapezoid.

Figure 4a is the circular grid map method used in [1], and Figure 4b is the trapezoidal grid map method proposed in this paper. A grid map is applied to recognize the surrounding environment of the vehicle and detect free space. As shown in Figure 4a, the grid map used in the previous paper is in the shape of a circle. In the circular grid map, the center of the circle is fixed in absolute coordinates, the radius of the circle is determined according to the speed of the driving vehicle, and the position of the vehicle on the circle is determined according to the heading of the vehicle. As the speed of the vehicle increases, the radius increases, and the vehicle’s position on the circle becomes opposite when moving forward and backward.

The newly proposed trapezoidal grid map is shown in Figure 4b. The height of the trapezoid is determined according to the speed of the vehicle, and the lower and upper sides of the trapezoid are determined according to the vehicle heading. As the speed is high and the heading is small, the trapezoid has a long and narrow shape, and if the speed is low and the heading angle is large, the trapezoid shows a short and wide shape. The trapezoidal shape is used because it can reduce the use of unnecessary cells and reflects the vehicle yaw angle better than the circular shape.

(1) $$US=|HD|\times a+b_{1}$$

(2) $$BS=|HD|\times a+b_{2}\;$$

(3) H=V/c+d

Equations (1)–(3) are equations for the proposed trapezoidal grid map generation. *US* is the upper side of the trapezoid(m), *BS* is the base of the trapezoid(m), and *H* is the height of the trapezoid(m). *HD* is the vehicle’s heading (deg), and *V* is the vehicle’s speed (kph).

*a*, $b_{1}$, $b_{2}$, *c*, and *d* are constant values. a is a constant value for converting the heading value expressed in deg units to the base of the trapezoid in m units, $b_{1}$ is the minimum value of the upper side of the trapezoid (*US*), and $b_{2}$ is the minimum value of the base of the trapezoid (*BS*). When the heading of the vehicle is zero, the length of the upper side *US* of the trapezoid is $b_{1}$, and the length of the base side *BS* is $b_{2}$.

(4) R=V× e+f

Equation (4) is a simplified formula for generating a prototype grid map used in previous papers. *R* is the radius of the circle (m) and *V* is the speed of the vehicle (kph). *e* and *f* are constant values. *e* is a constant value for converting the vehicle speed value expressed in kph units to the radius of the circle in m units, and *f* is the minimum value of the radius (*R*). The length of the *R* is f when the vehicle speed is zero.

### 2.3. Free Space Detection (FSD)

**Figure 5:**
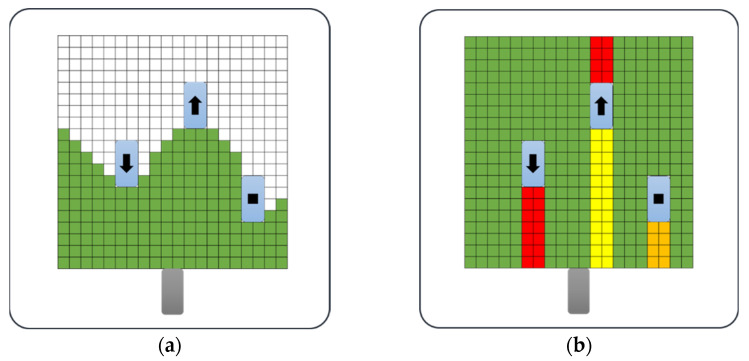
Free space detection with polygon and free space detection with segments: (**a**) polygon method of previous paper; (**b**) proposed segmentation method.

As shown in Figure 5, the free space is represented by segmentation. The authors of [1] used a method to represent free space by extracting free space boundary cells from the grid map, as shown in Figure 5a, and simplifying the polygons connecting the cells. Since this paper focuses on classifying the free space area according to the behavior of obstacles, it would be more appropriate to express it in a divided way rather than a polygon, as shown in Figure 5b.

Figure 5 shows the different free space detection methods. In the figure, the gray squares represent a vehicle in motion, and the three blue squares represent obstacles. The direction of movement of the obstacle is indicated by an arrow, and the obstacle drawn with a black rectangle instead of an arrow indicates that it is stationary. Figure 5a shows a free space detection method used in previous papers, representing free space with polygons. Figure 5b is the method proposed in this paper, which represents free space as a segment.

Figure 5b shows the separated free space area in color. It has a different meaning depending on the segment color. Green is the space where you can drive because there are no obstacles in front, yellow is the space where you can drive with obstacles, orange is where the obstacle is stationary or you are driving at a lower speed than the ego vehicle, meaning it is impossible to drive, and red is the direction the obstacle is moving. Conversely, if the vehicle is traveling at a high speed, it is impossible to drive, and there is a collision risk. Even if it is detected as a free space, the free space is detected and classified to reflect that there is a space where driving is impossible.

Segments are classified according to the perceived speed of the obstacle. If an obstacle is driving in the opposite direction to the vehicle being driven, there is a collision risk with the obstacle when creating a path and driving in this space, even if there is a free space in the direction of the obstacle. Conversely, if there is not enough free space due to the obstacle, but the obstacle is traveling in the same direction as the currently traveling vehicle and at a higher speed, this space will become a drivable space. As shown in Table 2 the free space is classified according to the driving possibility by dividing the cases where the behavior of the obstacles is different into four categories. In Table 2, the divided free space is represented by a total of four expressions: CA (completely able to drive), A (able to drive), UA (unable to drive), and CUA (completely unable to drive).

In Table 2, the relative speed of the obstacle (S) is the relative speed between the driving vehicle and the obstacle. First, if there are no obstacles in the segmented segment, the segment is divided into a completely drivable area (CA; completely able to drive). Second, if there is an obstacle in the segment, but the relative speed of the obstacle is greater than or equal to 0, it is classified as an able to drive area (A; able to drive). Even if there is an obstacle near the front, if the relative speed is greater than or equal to 0, the collision risk is low even if the vehicle continues driving without avoiding the obstacle. Third, when the relative speed of the obstacle is less than 0 and greater than the value obtained by multiplying the vehicle speed by -1, the area is classified as an unable to drive area (UA; unable to drive). This means that the obstacle and the vehicle are driving in the same direction, and the vehicle is driving faster than the obstacle. In this case, when driving in this segment, there is a collision risk with obstacles, meaning it is classified as an impossible area. Fourth, if the relative speed is less than the vehicle speed multiplied by −1, it is classified as a completely unable to drive area (CUA; completely unable to drive). This means that there is an obstacle moving in the opposite direction to the vehicle. That is, it represents an obstacle in a lane opposite to the vehicle driving lane. When driving in this segment, the vehicle runs in the opposite direction, meaning that even if there is a free space between the vehicle and an obstacle, it is divided into an area where driving is impossible.

In the pseudocode below (Algorithm 1), P denotes an obstacle, RS denotes the relative speed of the obstacle and the vehicle, VS denotes the speed of the vehicle, and Sp denotes the separation of free space.
**Algorithm 1.** Free Space Detection (P, RS, VS, Sp)
**for** each obstacle P  **if** P exist        calculation RS        **if** RS ≥ 0            mark Sp as able to drive        **else if** -VS < RS < 0            mark Sp as unable to drive        **else if** RS < -VS            mark Sp as completely unable to drive   **else**        mark Sp as completely able to drive

### 2.4. Path Generation

In order to reach a set target point, it is necessary to create an optimal path. The A* algorithm is used for path generation [32,33]. A weight is assigned to each cell of the grid according to the result of classifying the free space in which driving is possible and the space which cannot be driven in, and according to the distance from the target point. The weights of cells adjacent to the current location of the vehicle are calculated, and a path is generated to the cell with the smallest weight, that is, the lowest cost. By repeatedly executing this process, it is possible to generate the shortest optimal path considering the collision risk with obstacles.

**Figure 6:**
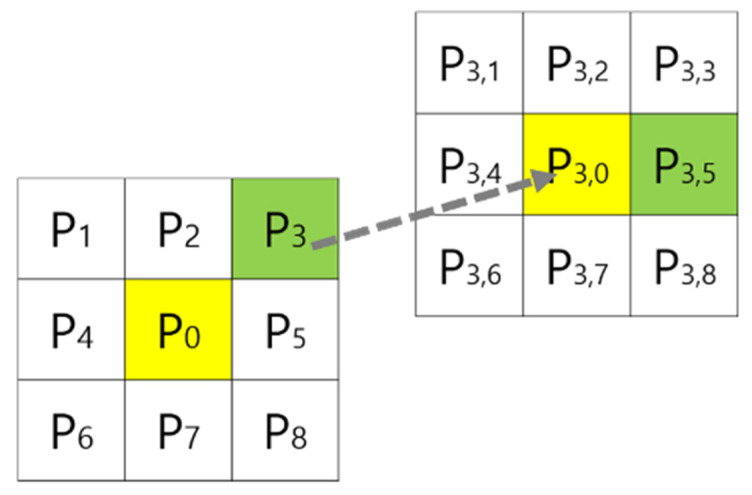
Selection of the cell with the higher weight among neighboring cells.

As shown in Figure 6, when the weight of the cell $P_{1}\cdots P_{8}$ is calculated at the current position $P_{0}$, if the cell with the lowest weight is $P_{3}$, the path is created in the direction from $P_{0}$ to $P_{3}$. In the next step, the weights of adjacent cells are calculated based on $P_{3}$, and then a path is created.

The weight for each divided area is calculated according to the calculation constant (*u*) in Table 3. The weight is the product of the linear distance to the target point multiplied by the calculation constant u. In the region where there is a high possibility of driving without obstacles, the smallest value is 1, and when the relative speed of the obstacle is greater than or equal to 0, the weight is 2. In the area where the relative speed of the obstacle is less than 0, but it is running in the same direction as the vehicle, the calculated constant value is 3, and the area where the obstacle is running in the opposite direction to the vehicle is set to 4.

### 2.5. Time to Collision and Collision Risk

To confirm that the proposed method (free space detection using speed information) is an improvement of the previous method (free space detection without speed information), the CR (collision risk) was calculated using TTC (time to collision) [34,35]. Two methods were used to create a path in the detected free space and calculate the CR when the path is driven. After taking the reciprocal of TTC, the reciprocal of TTC with all obstacles was added to the ego vehicle position and speed. Then, this value was added at all points in the generated path. The result of this calculation is the CR value.

In Equation (5), $d_{rel}$ is the distance between the position of the vehicle and the obstacle, and $v_{rel}$ is the relative speed of the vehicle and the obstacle [34]. In Equation (6), $o_{1}$…$o_{n}$ represent an obstacle, *n* is the number of obstacles, and the reciprocal of TTC corresponding to all obstacles is added. pt represents each point of the path, and it is the sum of all corresponding values from the starting point $pt_{0}$ of the path to the last point $pt_{m}$ of the path.

(5) $$TTC=\frac{\left|\vec{d_{rel}}\right|}{\left|\vec{v_{rel}}\right|\;cos\theta }$$

(6) $$CR=\overset{pt_{m}}{\underset{pt_{0}}{\sum }}(\overset{o_{n}}{\underset{o_{1}}{\sum }}\frac{1}{TTC})\;$$

### 2.6. Experiment Environments

**Figure 7:**
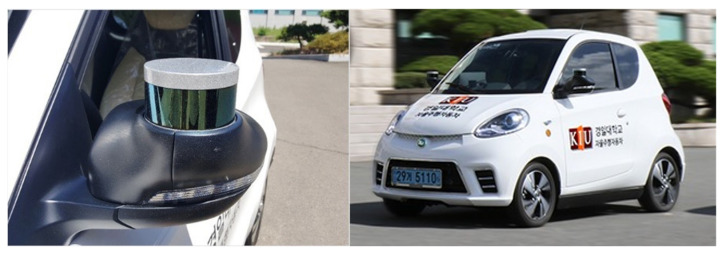
LiDAR sensor on the vehicle.

A LiDAR sensor was attached to a small electric vehicle, and the sensor data were measured while driving on a real road. Sensor data were acquired in various environments, such as static obstacles, dynamic obstacles, and obstacles in real road driving. The model was a Velodyne VLP-16. Table 4 shows the technical specifications of the LiDAR sensor used. Figure 7 shows the LiDAR sensor and vehicle used for data measurement. The vehicle used was an electric vehicle, D2, owned by Kyungil University, which has obtained an autonomous driving license from the Ministry of Land, Infrastructure and Transport, Korea.

Table 5 shows the constant values (*a*, $b_{1}$, $b_{2}$, *c*, *d*, *e*, *f*) of Equations (1)–(4).

## 3. Results 

**Figure 8:**
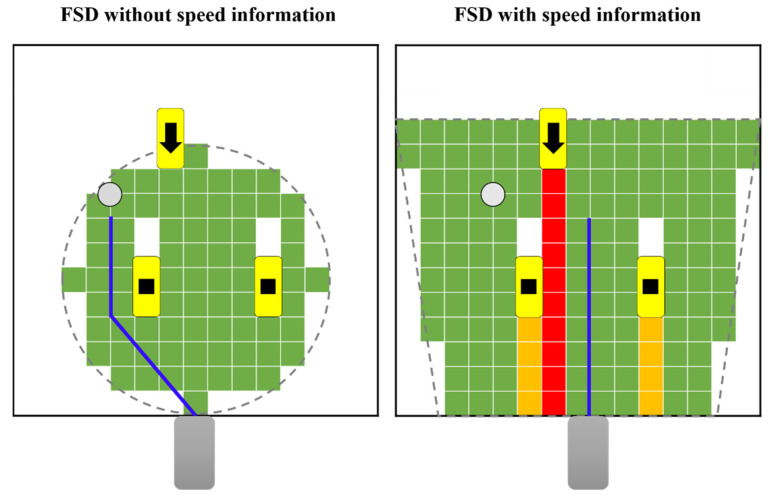
Pictorial result of path generation based on FSD of two methods in case Figure 9a.

**Figure 9:**
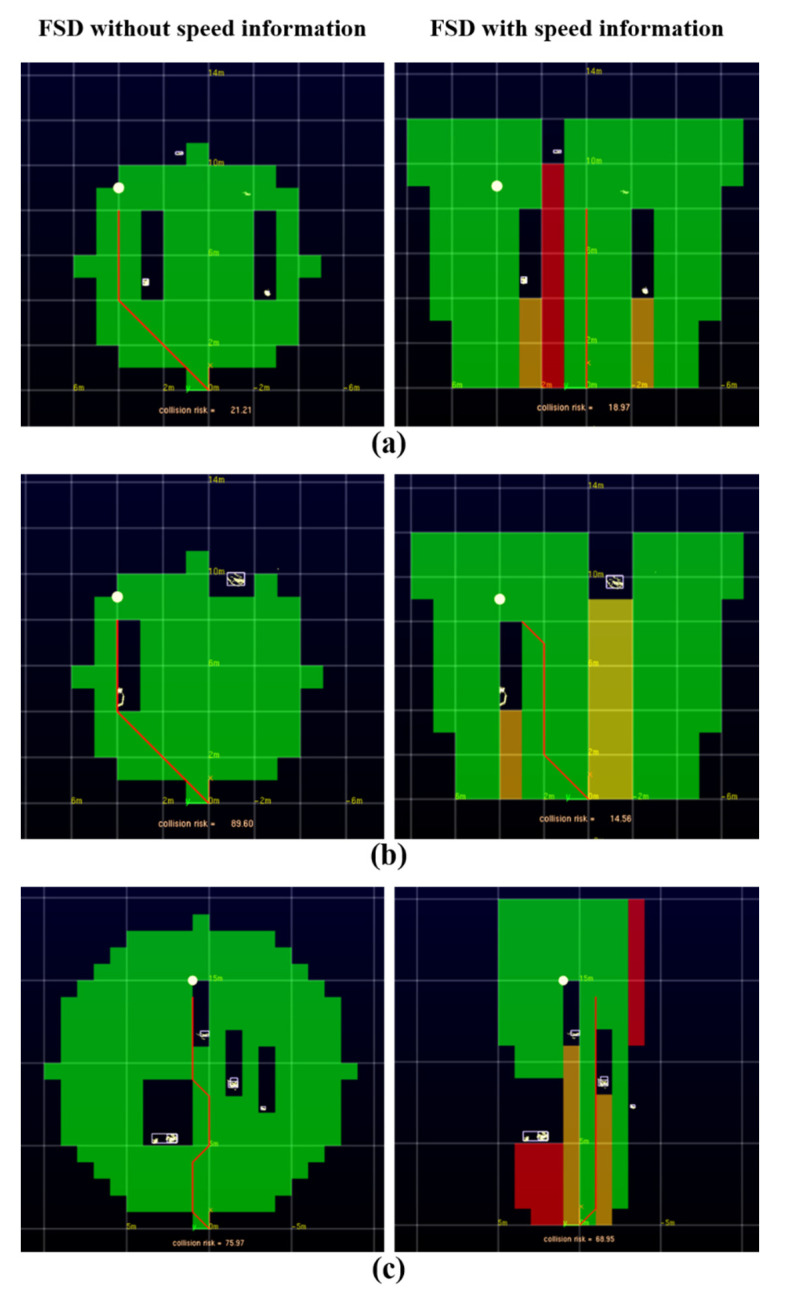

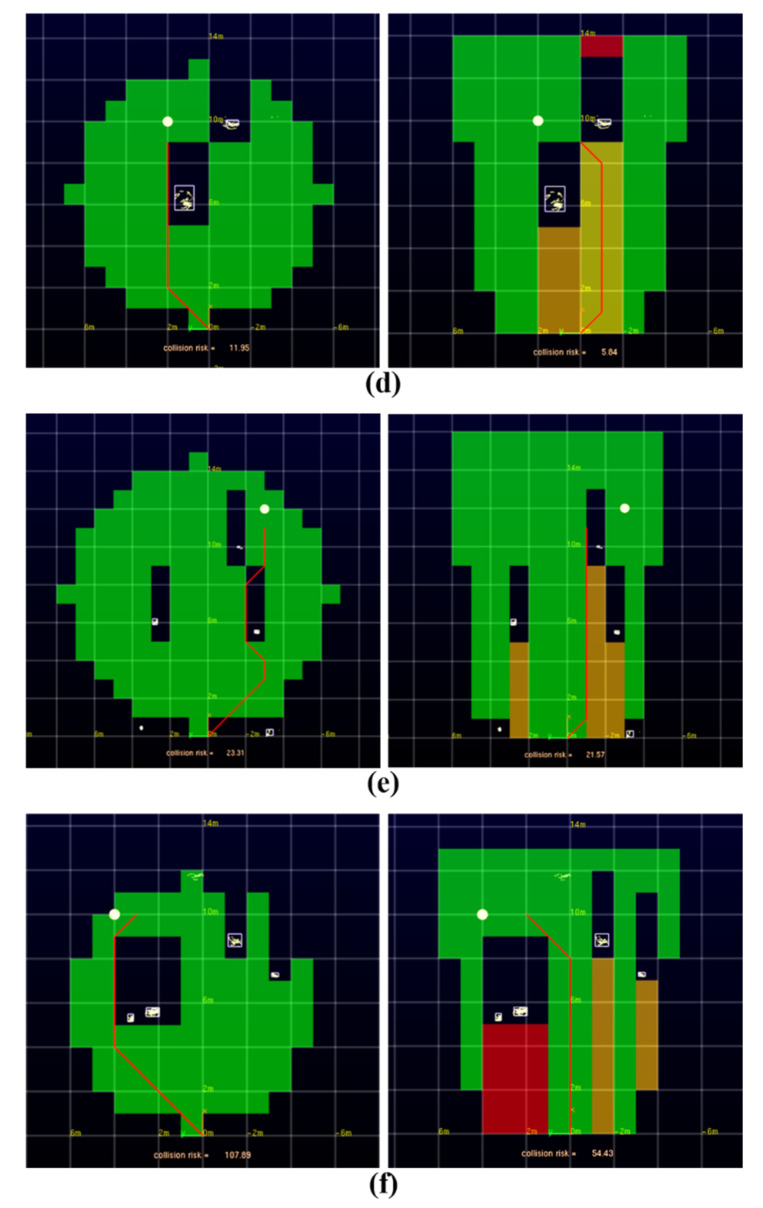
Generated path comparison of FSD without speed information in circular grid map and FSD with speed information in trapezoid grid map in cases (**a**)–(**f**).

Figure 8 is a pictorial representation of the results of Figure 9a. In Figure 8, green is the free space that the vehicle can drive in. In the figure, red is a space in which the vehicle is completely unable to drive, and orange is a space in which the vehicle is unable to drive. The dotted line represents the grid map range, and the yellow represents the obstacle. The direction in which the obstacle moves is represented by an arrow, and the static obstacle is represented by a square dot. The driving target point is indicated by a gray circle. Paths are generated only in free space cells, and the generated paths are indicated by blue lines. By referring to this pictogram, the execution result screen of Figure 9 can be grasped. 

Figure 9 show the execution screen of generating a path based on the FSD of the two methods in six cases. Figure 9 and Table 6 are the FSD results in cases (a)–(f). Case (a) shows one dynamic obstacle moving in opposite directions and two static obstacles. Case (b) has one dynamic obstacle moving in the same direction and one static obstacle. Case (c) is a case with two dynamic obstacles moving in opposite directions and two static obstacles. Case (d) has one dynamic obstacle moving in the same direction and one static obstacle. Case (e) is a case with three static obstacles. Case (f) is a case with two dynamic obstacles moving in opposite directions and two static obstacles.

Figure 9 are the results of the comparison between the cases of FSD without obstacle speed information in the circular grid map and the case of FSD by dividing areas with speed information of obstacles in the trapezoidal grid map. In Figure 9, the white point is the vehicle driving target point, and the red line represents the generated path according to the free space. As a result of FSD without speed information, it can be seen that a path with a collision risk is created even though there is an obstacle moving in the direction opposite to the vehicle direction. In the result of FSD with speed information, it is determined that the area where the obstacle is moving in the opposite direction to the vehicle’s driving direction is an impossible area, and it is divided into red areas to indicate that a safe path is created in the space, not in the driving direction of the obstacle.

Table 6 is a quantitative representation of the comparison results of the two cases. As a result of the calculation, the results shown in Table 6 were obtained. A smaller CR value means that there is less risk of colliding with obstacles around the vehicle when traveling on that path. When the free space is detected using the speed information, the CR value is smaller than when the speed information is not used, and more secure driving is possible.

## 4. Discussion

The purpose of this study was to ensure the stable operation of autonomous vehicles. While it is important for autonomous vehicles to travel on the shortest route, it is also important to drive on a safe and optimal route with less collision risk. Most obstacles surrounding a moving vehicle are dynamic obstacles. Therefore, speed information must be taken into account when detecting free space. By excluding the free space in which driving is impossible in consideration of the speed information, unnecessary calculations can be reduced, and the risk of a collision can be further reduced. This can be confirmed by the difference between the result path creation screen and the collision risk. If this algorithm is applied to an autonomous vehicle, a safe route can be generated in various environments where route creation is impossible, such as an environment where lanes are not recognized, or where it is difficult to build an accurate map. Therefore, safer driving is possible through this algorithm.

## 5. Conclusions

In autonomous driving, generating the fastest path is important, but creating a safe path with less collision risk with surrounding obstacles and driving safely are also two of the most important aspects. As a result of the path generation comparison, when the FSD algorithm including the speed information proposed in this paper was used, it was possible to generate a path with a low collision risk. In the future, we plan to conduct research on generating a path in real time in a more complex driving environment, avoiding obstacles by controlling the actual vehicle according to the path, and autonomously driving to the destination.

## Figures and Tables

**Table 1:** Summary of previous papers [1,2,3,4,5,6,7,8,9,10,11,12].

Previous Paper	Sensor	FSD with/without Obstacle Speed Information
[1]	Laser Scanner	without
[2]	LiDAR, Camera	
[3]	LiDAR	
[4]	LiDAR, Camera	
[5]	Camera	
[6]	Camera	
[7]	LiDAR, Camera	
[8]	Camera	
[9]	Camera	
[10]	Radar	
[11]	Camera	
[12]	Camera	

**Table 2:** Driving possibility according to object speed.

Relative Speed of the Obstacle (S)	Possibility of Driving
No obstacles	CA
S ≥ 0	A
-(vehicle speed) < S < 0	UA
S < -(vehicle speed)	CUA

**Table 3:** Weight according to free space classification.

Possibility of Driving in a Demarcated Area	*u*
CA	1
A	2
UA	3
CUA	4

**Table 4:** Velodyne VLP-16 specifications.

Specifications	Value
Channel	16 channels
Measuring range	100 m
Accuracy	Max ±3 cm
Field of view (vertical)	+15.0° to −15.0°
Field of view (horizontal)	360°
Angular resolution (vertical)	2.0°
Angular resolution (horizontal/azimuth)	0.1–0.4°
Rotation speed	5–20 Hz

**Table 5:** Constant values of Equations (1)–(4).

Constant	Value
*a*	0.46
$b_{1}$	6
$b_{2}$	2
*c*	4.3
*d*	2
*e*	0.1
*f*	1

**Table 6:** Collision risk (CR) in cases (**a**)–(**f**).

Case	CR	
	FSD without Speed Information	FSD with Speed Information
(a)	21.21	18.97
(b)	89.60	14.56
(c)	75.97	68.95
(d)	11.95	5.84
(e)	23.31	21.57
(f)	107.89	54.43
